# Protective Efficacy of the Trivalent *Pseudomonas aeruginosa* Vaccine Candidate PcrV-OprI-Hcp1 in Murine Pneumonia and Burn Models

**DOI:** 10.1038/s41598-017-04029-5

**Published:** 2017-06-21

**Authors:** Feng Yang, Jiang Gu, Liuyang Yang, Chen Gao, Haiming Jing, Ying Wang, Hao Zeng, Quanming Zou, Fenglin Lv, Jinyong Zhang

**Affiliations:** 1 College of Bioengineering, Chongqing University, Chongqing, 400030 PR China; 2 National Engineering Research Center of Immunological Products, Department of Microbiology and Biochemical Pharmacy, College of Pharmacy, Third Military Medical University, Chongqing, 400038 PR China

## Abstract

*Pseudomonas aeruginosa* is a formidable pathogen that is responsible for a diverse spectrum of human infectious diseases, resulting in considerable annual mortality rates. Because of biofilm formation and its ability of rapidly acquires of resistance to many antibiotics, *P*. *aeruginosa* related infections are difficult to treat, and therefore, developing an effective vaccine is the most promising method for combating infection. In the present study, we designed a novel trivalent vaccine, PcrV_28-294_-OprI_25-83_-Hcp1_1-162_ (POH), and evaluated its protective efficacy in murine pneumonia and burn models. POH existed as a dimer in solution, it induced better protection efficacy in *P*. *aeruginosa* lethal pneumonia and murine burn models than single components alone when formulated with Al(OH)_3_ adjuvant, and it showed broad immune protection against several clinical isolates of *P*. *aeruginosa*. Immunization with POH induced strong immune responses and resulted in reduced bacterial loads, decreased pathology, inflammatory cytokine expression and inflammatory cell infiltration. Furthermore, *in vitro* opsonophagocytic killing assay and passive immunization studies indicated that the protective efficacy mediated by POH vaccination was largely attributed to POH-specific antibodies. Taken together, these data provided evidence that POH is a potentially promising vaccine candidate for combating *P*. *aeruginosa* infection in pneumonia and burn infections.

## Introduction

*Pseudomonas aeruginosa* is one of the leading formidable pathogens responsible for hospital-acquired pneumonia, surgical infection, bacteremia and other life-threatening infections worldwide^1^. It is especially problematic in long-term hospitalized patients in intensive care units and burn victims. *P*. *aeruginosa* causes ventilation-associated pneumonia and contributes to a mortality rate as high as 13.5%^2^. *P*. *aeruginosa* is the most frequent Gram-negative pathogen in burn patients, and infection with this pathogen can quickly lead to a systemic infection with a mortality rate ranging from 38% to 70%^3^. Conventional use of antibiotics for the treatment of *P*. *aeruginosa* infections is of restricted efficacy, and the emergence of antibiotic-resistant bacteria makes the treatment of *P*. *aeruginosa* infection a persistent problem^4^. To combat the spread of *P*. *aeruginosa* infection, an effective vaccine or other alternative immunotherapy is urgently needed.

Several *P*. *aeruginosa* vaccine candidates have been tested^5–9^, however, no vaccine has yet been licensed for clinical use^10^. One great challenge for the development of vaccines against *P*. *aeruginosa* is that the bacteria utilize multiple pathways to cause infection, and vaccines that block only one or two of the pathways are unable to provide effective protection^11^. In addition, frequent variations in the *P*. *aeruginosa* genome make selecting conserved antigens difficult^12^. *P*. *aeruginosa* attaches to host cells *via* flagella, type 4 pili and other adhesion sites, additionally, the bacteria use dozens of proteases to cause tissue damage. Moreover, *P*. *aeruginosa* encodes a type III secretion system (T3SS) and a type VI secretion system (T6SS), which directly inject effector molecules into host cells and disrupt cellular functions^11^.

The *P*. *aeruginosa* V-antigen (PcrV) is an extracellular component of the T3SS that enables killing of epithelial and immune cells by protein toxin injection^13^. Promising results from a phase II trial of KB001, a recombinant anti-PcrV Fab fragment, showed that it reduced airway inflammation and damage in CF patients with chronic *P*. *aeruginosa* infection^14^. Outer membrane protein I (OprI) is a major surface lipoprotein that plays a vital role in bacterial susceptibility to antimicrobial peptides^15, 16^. The most promising *P*. *aeruginosa* vaccine (IC43), which was evaluated in a phase III clinical trial (NCT01563263), is composed of OprI and a fragment of the outer membrane protein OprF. In addition, OprI acts as an adjuvant by triggering the TLR2/TLR4 pathway to improve immunity against *Mycobacterium tuberculosis* and classical swine fever^17–19^. Hemolysin co-regulated protein 1 (Hcp1) is a central component of the T6SS-dependent intercellular effector transport which can deliver toxin proteins directly into prey prokaryotes and also eukaryotic cells during bacterial infection^20^.

After a critical review of the immunogenicity, distribution and crucial role of PcrV, OprI and Hcp1 in *P*. *aeruginosa* pathogenesis, we hypothesized that a combination of these proteins could induce a significant immune response and provide full protection against infection. In this study, we generated a trivalent vaccine containing PcrV, OprI and Hcp1 and evaluated its immunogenicity and protective potential in an acute pneumonia and burn mouse model.

## Methods

### Ethics statement

All animal care and use protocols in this study were performed in accordance with the Regulations for the Administration of Affairs Concerning Experimental Animals approved by the State Council of People’s Republic of China. All animal experiments in this study were approved by the Animal Ethical and Experimental Committee of the Third Military Medical University (Chongqing, Permit No. 2011-04) in accordance with their rules and regulations. All surgery was performed under sodium pentobarbital anesthesia, and all efforts were made to minimize suffering.

### Bacterial strains and culture method

The *P*. *aeruginosa* standard strain PAO1 was purchased from ATCC (Manassas, VA, USA). Clinical strains of 4 *P*. *aeruginosa* isolates were collected from 4 hospitals in different districts of China (Supplementary Table S1). Bacterial strains were cultured in Luria Bertani broth, washed and diluted with sterile PBS to an appropriate cell concentration determined spectrophotometrically at 600 nm (OD_600_).

### Animals

8 to 12-week-old female BALB/c mice were purchased from Beijing HFK Bioscience Limited Company (Beijing, People’s Republic of China). Mice were matched for age and sex, and kept under specific pathogen-free (SPF) conditions. Female New Zealand white rabbits (weighing 2.00 ± 0.20 kg) were provided by TengXin Company (Chongqing, China).

### Cloning, expression and purification of recombinant proteins

**Figure 1:**
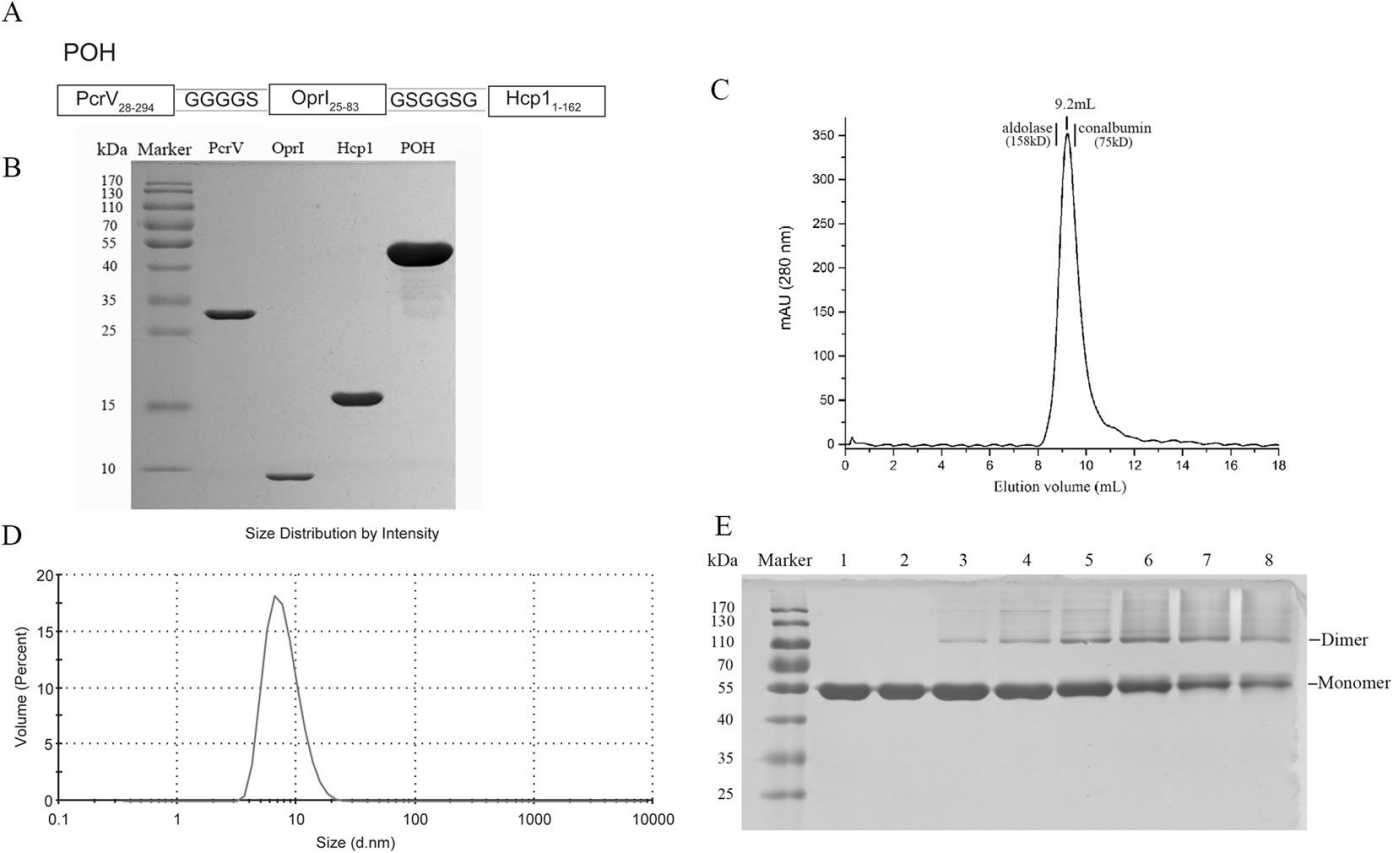
Preparation and characterization of antigens. (**A**) Schematic diagram illustrating the primary structure of POH. (**B**) PcrV, OprI, Hcp1 and POH were purified and analyzed by SDS-PAGE. (**C**) Oligomeric states of POH determined by size elution chromatography. The elution volumes for POH, protein standard aldolase (158 kDa) and conalbumin (75 kDa) were 9.2 ml, 8.8 ml and 9.6 ml, respectively. (**D**) Dynamic light scattering analysis of POH demonstrated that POH formed a symmetrical peak with a diameter of 6.9 nm, and the estimated molecular weight was approximately 91.2 ± 22.4 kDa. (**E**) SDS-PAGE analysis of POH after treatment with different concentrations of glutaraldehyde. Lane 1 shows the native POH. Lanes 2 to 8 show the formation of dimers or aggregates with the increasing concentration of glutaraldehyde (0.01%, 0.05%, 0.1%, 0.2%, 0.3%, 0.4%, and 0.5%).

The sequences encoding PcrV (28–294), OprI (25–83) and Hcp1 (1–162) were amplified from the genome of *P*. *aeruginosa* PAO1 and cloned into an expression vector derived from the pGEX-6P-2 plasmid and placed between the *Bam*HI and *Xho*I restriction sites. The primers used for gene amplification are listed in Supplementary Table S2. The N-terminus of each protein was fused to a Glutathione S-transferase (GST) tag to facilitate protein expression and purification. The DNA sequence encoding recombinant PcrV-OprI-Hcp1 (POH) was synthesized and inserted into the pGEX-6P-2 vector between the same restriction sites by Sheng Gong Biological Engineering (Shanghai, China). The POH DNA and amino acid sequences are shown in Supplementary Table S3, and two linkers, “GGGGS” and “GSGGSG,” were inserted among the three proteins (Fig. 1A). For expression of recombinant proteins, these vectors were transformed into the *E*. *coli* strain XL-1 Blue (Biovector Science Lab, Beijing, China), and the bacteria were grown in LB media supplemented with ampicillin (100 μg/ml) until the optical density at 600 nm (OD_600_) reached 0.6. Isopropyl β-D-1-thiogalactopyranoside (IPTG) was then added to a final concentration of 0.2 mM to induce expression of the recombinant protein at 16 °C overnight.

All four recombinant proteins were purified according to the same protocol. In brief, the cells were harvested by centrifugation, and the bacterial pellets were resuspended in PBS and disrupted by ultrasonication. Insoluble cellular material was removed by centrifugation, and the supernatant was mixed with Glutathione Sepharose 4B agarose (GE Healthcare, Piscataway, NJ, USA). After washing the beads 4 times with PBS and adding preScission protease (GE) to the mixture to cleave the N-terminal GST tag, the supernatant containing the GST-free protein was collected and concentrated to approximately 1.0 ml and applied onto a HiLoad 16/60 Superdex 75 prep-grade gel-filtration column (Amersham Biosciences) equilibrated with His buffer (0.01 M His, 0.15 M NaCl, pH 6.0). The peak fractions from size exclusion analysis that corresponded to the recombinant protein were pooled. Endotoxin was further removed by Triton X-114 phase separation as described elsewhere^21^. Protein purity was determined by SDS-PAGE and high-performance liquid chromatography (HPLC). Protein concentration was determined using the bicinchoninic acid (BCA) method (Pierce). The endotoxin content was detected using the tachyplens ameboyto lysate assay (Houshiji cod Inc., Xiamen, China). And endotoxin levels were <2.5 pg/μg for all the four recombinant proteins^22^.

### Gel-filtration and dynamic light scattering assay

The oligomeric states of the fusion protein POH was determined by gel-filtration chromatography using Superdex^TM^ 75 10/300 GL based on a method established by us previously^23^. Gel Filtration Calibration Kits (GE Healthcare, Piscataway, NJ, USA) was used to generate the calibration curve, 200 μl purified POH was loaded onto the column, and the elution volume of the corresponding peak was used to calculate the molecular weight, thereby determining the oligomeric state of the protein. For dynamic light scattering assay, POH was diluted to 0.5 mg/ml and loaded onto a dynamic light-scattering instrument (Zetasizer) equipped with an argon ion laser, the analysis was performed three times at 25 °C.

### Chemical cross-linking analysis

We followed the protocol described by Fadouloglou previously^24^. In brief, POH was diluted to 1 mg/ml and incubated with glutaraldehyde at 37 °C for 30 min. The final concentration of glutaraldehyde in each reaction was 0.01%, 0.05%, 0.1%, 0.2%, 0.3%, 0.4%, and 0.5%. The cross-linking reaction was then terminated by adding loading buffer containing SDS and glycine. The protein samples were then analyzed by SDS-PAGE.

### Immunization of the mice

For active immunization, purified proteins in His buffer were emulsified 1:1 (volume ratio) in Freund’s (Sigma), MF59 (Invivogen), Al(OH)_3_ (Pierce), AlPO_4_ (Pierce), or AS04 (GSK). Mice were intramuscularly injected with 200 μl of the emulsion containing 30 μg protein, His buffer plus adjuvant, or His buffer alone as the control on days 0, 14, and 21. And, mice were infected at day 28.

For passive immunization, polyclonal antibodies (pcAb) were generated in rabbits based on a previously published method^25^. The IgG in the serum from antigen immunized or unimmunized rabbits were purified by affinity chromatography with a protein A column (GE, USA), followed by desalting with PBS, the concentration of each pcAb was determined by the BCA method^26^ and adjusted to a final concentration of 20 mg/ml. Two hours before infection, mice were injected intravenously with 2 mg of each pcAb, 2 mg of negative control pcAb or the same volume of PBS.

### *P. aeruginosa* infection

For acute pneumonia model, mice in each group were anesthetized with pentobarbital sodium followed by intracheal injection of a lethal dose of *P*. *aeruginosa* isolates to measure the survival rates. The number of deaths in each group was recorded every 12 hours in a 7-day observation period post-challenge. The lethal dose of *P*. *aeruginosa* strains PAO1, XN-1, BJ-15, GZ-18 and KM-9 were determined to be 1.0 × 10^7^, 6.0 × 10^6^, 2.0 × 10^7^, 2.0 × 10^7^ and 4.0 × 10^6^ colony-forming units (CFUs), respectively. For bacterial burdens, histopathology, inflammatory cells and cytokines analyses, mice were infected intracheal injection with 5.0 × 10^6^ CFUs of PAO1. In sub-lethal challenge group, the overall health condition of animals was assessed by piloerection, posture, locomotion, breathing rate and nasal secretion, resulting in the following global disease score: unaffected (0–1), slightly affected (2–4), moderately affected (5–7), severely affected (8–10)^27^.

In burn model, mice were burned and challenged as described previously^28^. Briefly, the anesthetized, shaved mouse was placed supine in a device and immersed in boiling water (88 °C). A burn wound (12–15% of the body surface area, third-degree) was created after ten seconds of exposure. Immediately after burning, 0.5 ml of sterile saline solution was injected intraperitoneally into the mice for fluid replacement. Acetaminophen (0.25 mg/ml) was used as post-burn analgesic. Next, the mice were infected by subcutaneous injection of *P*.*aeruginosa* strains PAO1, XN-1, BJ-15, GZ-18 and KM-9 (300 CFUs) at the burn site, respectively. 24 h after subeschar infection, bacterial burdens were determined, and survival rate of experimental mice was monitored for 7 days.

### ELISA

On day 28 after primary immunization, mice were exsanguinated, and serum samples were collected for the enzyme-linked immunosorbent assay (ELISA). Wells of microtiter plates (Thermo Labsystems) were coated with PcrV, OprI, Hcp1 or POH (400ng per well) in 0.05 M carbonate buffer (pH 9.5) overnight at 4 °C. Diluted serum samples were used as the primary antibodies. The secondary antibodies were HRP-conjugated goat anti-mouse IgG (Tianjin Sungene Biotech Co., Ltd., Tianjin, China), anti-IgG1, anti-IgG2a or anti-IgG2b (Sigma). The optical density at 450 nm was measured, and the titers were defined as the highest dilution that yielded an absorbance value of more than twice the value of the pre-immune serum.

### Aluminum adjuvant absorption capacity

As previously described^29^, AlPO_4_ and Al(OH)_3_ were diluted with His buffer to a final concentration of 1 mg/ml aluminum and then incubated with a gradient concentration of POH (0.2 to 2.0 mg/ml). The samples were mixed for 1 hour and then centrifuged. The concentration of unabsorbed protein in the supernatant was determined using the BCA assay. The assay was performed three times, and the adsorption capacity of the aluminum adjuvant was calculated according to the POH content in the supernatant.

### Bacterial burden

For pneumonia model, lung tissues were collected, weighed, and homogenized in 1 ml of sterilized PBS buffer 8 and 24 hours post-challenge. For burn model, 5 mice per group were sacrificed at 24 h after subeschar infection. To determine the local spread of the challenged bacterial, about 15 × 15 mm skin sections were removed from the burned skin of infected mice at the injection/inoculation site. To determine the systemic spread, liver, spleen, and blood samples were extracted from each animal. Individual skin sections and internal organs were weighed and homogenized in PBS. All homogenates were then plated on LB plates at a 10-fold serial dilution and cultured at 37 °C for 20 hours. Number of CFUs per gram of tissue (CFUs/g) was calculated from each plate.

### Histological analysis

Lung tissues were collected 24 hours post-infection from the pneumonia model mice. And skin sections were obtained 24 hours post-burned from the burn model. Lung and skin samples were fixed in neutral 10% formalin, embedded in paraffin, sectioned, and stained with hematoxylin and eosin (HE). The sections were then viewed at 100× magnifications by a single pathologist. Each lung section was given a score of 0–4 (no abnormality to most severe) according to established criteria based on hyperemia, edema, hemorrhage, and neutrophil infiltration^22^.

### Evaluation of inflammation

To quantify the neutrophils infiltration, cells in BALF from mice 24 hours post challenge were collected and stained using the following antibodies: PE/Cy7 anti-mouse CD45 and APC/cy7 anti-mouse Ly-6G (Biolegend, Inc, USA). Samples were then analyzed using BD FACSArray softwareTM on a BD FACS Array flow cytometer (BD Biosciences).

To quantify pro-inflammatory responses, cytokines such as TNF-α, IL-1β and IL-6 in BALF were collected 8 and 24 hours after infection with PAO1. The concentrations of pro-inflammatory cytokines were determined using a Mouse Quantikine ELISA kit for TNF-α, IL-1β or IL-6 (R&D Systems, USA), respectively, according to the manufacturer’s instructions.

### Cell proliferation and cytokine assays

Lymphocyte proliferation was determined using the cellular incorporation of 5-bromo-2-deoxyuridine (BrdU, Roche, Germany) and measured by absorbance at 450 nm^30^, and the stimulation index (SI) was calculated by dividing the absorbance of stimulated cells by absorbance of unstimulated cells. The lymphocyte proliferation rates and the phenotype of the proliferative cells was evaluated by using the intracellular fluorescent dye 5-(and -6)-carboxyfluorescein diacetate succinimidyl ester (CFSE) followed by Flow cytometric analysis^31^. APC/Cy7 anti-mouse CD3 Antibody (17A2, Biolegend), PerCP/Cy5.5 anti-mouse CD4 Antibody (RM4-4, Biolegend), APC anti-mouse CD8a Antibody (53−6.7, Biolegend) and PE anti-mouse CD19 Antibody (1D3/CD19, Biolegend) were used for cells staining. Cells treated with 10 mM phytohemagglutinin-A (PHA) (Gibco, USA) or not served as positive and negative controls, respectively. The levels of IFN-γ, IL-4 and IL-17 were measured in 72 hours cultures by examining cell-free culture supernatant fluid using a Mouse Quantikine ELISA kit for IFN-γ, IL-4 or IL-17 (R&D Systems, USA), respectively.

### Indirect immunofluorescence

Indirect immunofluorescence was carried out based on a method established by us previously^32^.

### Opsonophagocytic killing assay

The antibody opsonophagocytic killing assay was carried out as described by Burton and Nahm^33^. Briefly, HL-60 cells (ATCC, CCL-240) were differentiated into granulocyte-like cells in growth medium containing 100 mM N’,N-dimethylformamide (Sangon, Shanghai, China) for 5 days. Sera samples from immunized mice containing opsonic antibodies were heat-inactivated (56 °C, 30 min) and serially diluted with opsonization buffer (mix of 80 ml of sterile water, 10 ml of 10× Hank’s balanced salt solution, 10 ml of 1% gelatin, and 5.3 ml of fetal bovine serum). The assay was performed in 96-well plates, with each well containing the following components: 40 μl of 4 × 10^5^ HL60 cells, 10^3^ CFUs of PAO1 in 10 μl of opsonophagocytic buffer, 20 μl of serum, and 10 μl of 1% infant rabbit serum as a complement source (Pel-Freez). After a 2-h incubation, 10 μl of each sample was plated on agar medium. This experiment was performed in triplicate for each sample. The opsonophagocytic killing effect was defined as a reduction in CFUs after overnight growth compared with the CFUs in the sera from unimmunized mice.

### Statistical analysis

Data are presented as means ± Standard Deviation (SD) or means ± Standard Error of Mean (SEM). Scoring experiments were performed in a blind manner. Survival data were analyzed using Kaplan–Meier survival curves. To calculate *P* values, nonparametric Mann-Whitney test, log-rank test, Student’s t-test, one-way ANOVA with Bonferroni correction were used depending on sample distribution and variation as mentioned in figure legends. SPSS 13.0 (SPSS, Inc, USA) and GraphPad Prism 6.0 (GraphPad Software, Inc., USA) were used to perform statistical analyses. Significance was accepted at *P* < 0.05.

## Results

### POH is soluble and exists as a dimer in solution

As shown in Fig. 1B, all 4 proteins (PcrV, OprI, Hcp1 and POH) expressed in *E*. *coli* were soluble, and the purity was up to 95% as determined by SDS-PAGE after two rounds of chromatography. The molecular weights of these recombinant proteins were in accordance with their predicted molecular masses (29.6, 6.4, 17.4, and 54.1 kDa for PcrV, OprI, Hcp1 and POH, respectively).

The oligomeric state and homogeneity of recombinant proteins are of great importance for vaccine development because of their impact on antigen immunogenicity. As shown in Fig. 1C, the elution volumes for POH and the protein standard aldolase (158 kDa) and conalbumin (75 kDa) were 9.2 ml, 8.8 ml and 9.6 ml, respectively. The molecular weight of POH was calculated as 108.2 kDa. Additionally, POH formed a single peak when detected by dynamic light scattering (Fig. 1D). The size of the POH particle was approximately 6.9 ± 1.7 nm, and thereby, the estimated molecular weight was approximately 91.2 ± 22.4 kDa. The theoretical molecular weight of POH is 54.1 kDa, and thus, it appears to be a dimer in solution. This was further confirmed by a cross-linking assay, where a band corresponding to dimerized POH was observed on the gel in the presence of the glutaraldehyde, and the amount of the linked POH dimer increased in a glutaraldehyde dose-dependent manner (Fig. 1E).

### Al(OH)_3_ is an optimal adjuvant for POH immunization

**Figure 2:**
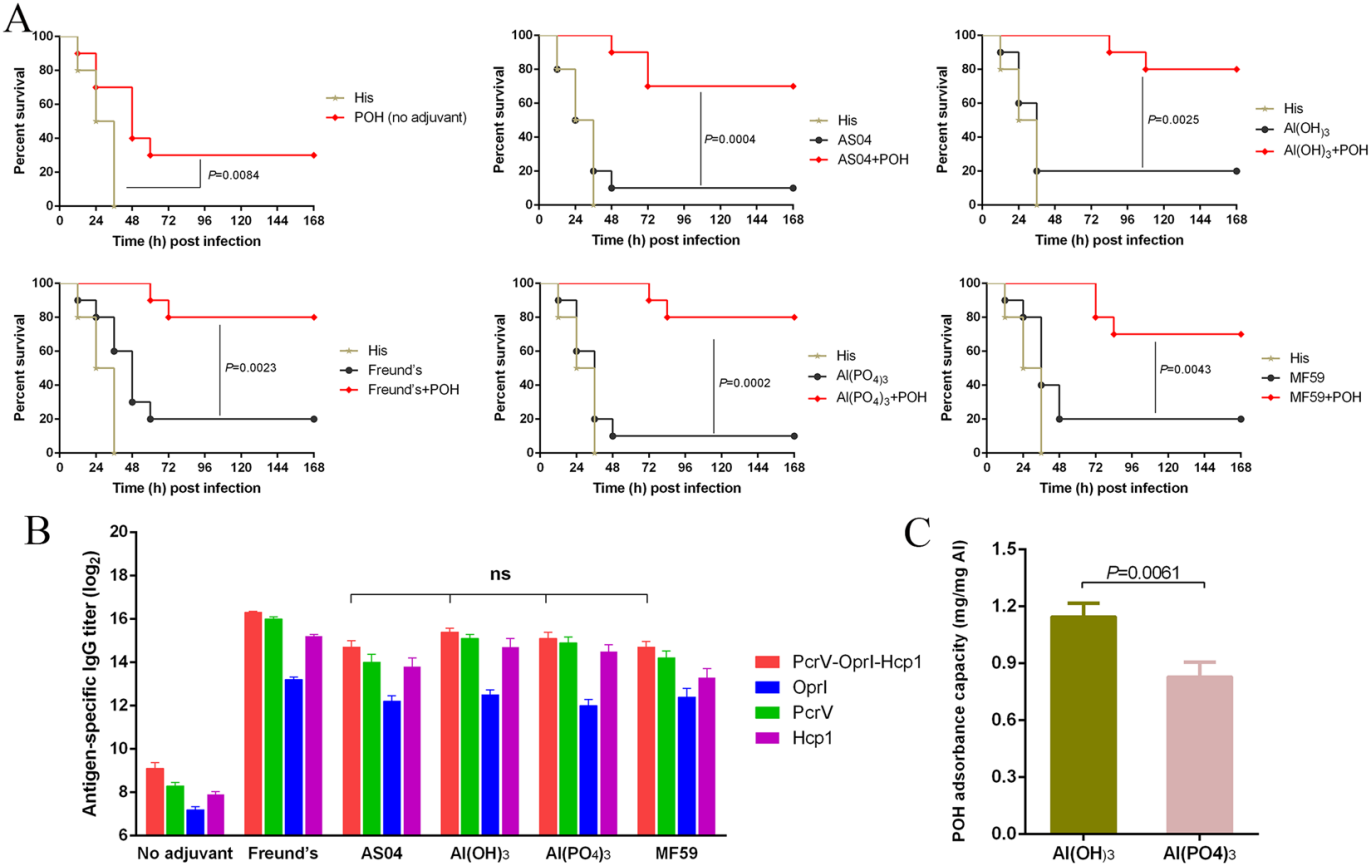
Validation of the optimal adjuvant for the recombinant protein POH in the pneumonia model. (**A**) BALB/c mice (n = 10) were immunized with the same doses of POH or POH plus different adjuvants (Freund’s, AS04, Al(OH)_3_, AlPO_4_ and MF59). The control groups included the adjuvant groups and the His buffer group. All of the mice were challenged with *P*. *aeruginosa* strain PAO1 at 1.0 × 10^7^ CFUs/mouse by intratracheal injection, and mouse survival was monitored for 1 week. The *P*-value was compared with the adjuvant control group using the Mantel-Cox log-rank test. (**B**) Plates were coated with POH, OprI, PcrV, and Hcp1, respectively. And serum was collected from mice (n = 10) in (**A**) immunized with POH or POH plus different adjuvants (Freund’s, AS04, Al(OH)_3_, AlPO_4_ and MF59) on day 28 before challenge. Antibody responses were expressed as the mean of log_2_ titers. Multiple comparisons among different groups were analyzed using one-way ANOVA (ns = no significance). Data were shown as the mean ± SEM. (**C**) Adsorption capacity of aluminum adjuvant. Student’s t-test was used to compare values. Data were shown as the mean ± SD.

To choose an optimal adjuvant for POH immunization, mice were immunized with POH formulated with different adjuvants (Freund’s, AS04, MF59, Al(OH)_3_ and AlPO_4_). The immunized mice were then challenged with lethal dose of PAO1 to evaluate the differences in protective efficacy. The survival rate of mice vaccinated with adjuvant-free POH was 30%, suggesting that vaccination with recombinant POH alone was able to provide partial protection (Fig. 2A). Further, improved survival rates were observed when an adjuvant is formulated with the fusion protein, which emphasis the importance of adjuvant for enhancing of protective efficacy (Fig. 2A). Furthermore, the titers of POH-, PcrV-, OprI-, and Hcp1-specific antibodies were not significantly different among the four adjuvant groups (Fig. 2B). Moreover, adjuvant absorb assay showed that Al(OH)_3_ exhibited a higher absorbance capacity of POH than did AlPO_4_ (Fig. 2C). As Freund’s adjuvant is not recommended for use in humans, and no significant difference in the level of antigen-specific antibodies and protection efficiency was observed among the other adjuvants, Al(OH)_3_ was chosen as the optimal adjuvant for the subsequent studies.

### POH vaccination protects mice from pneumonia by reducing bacterial burden and inflammation

**Figure 3:**
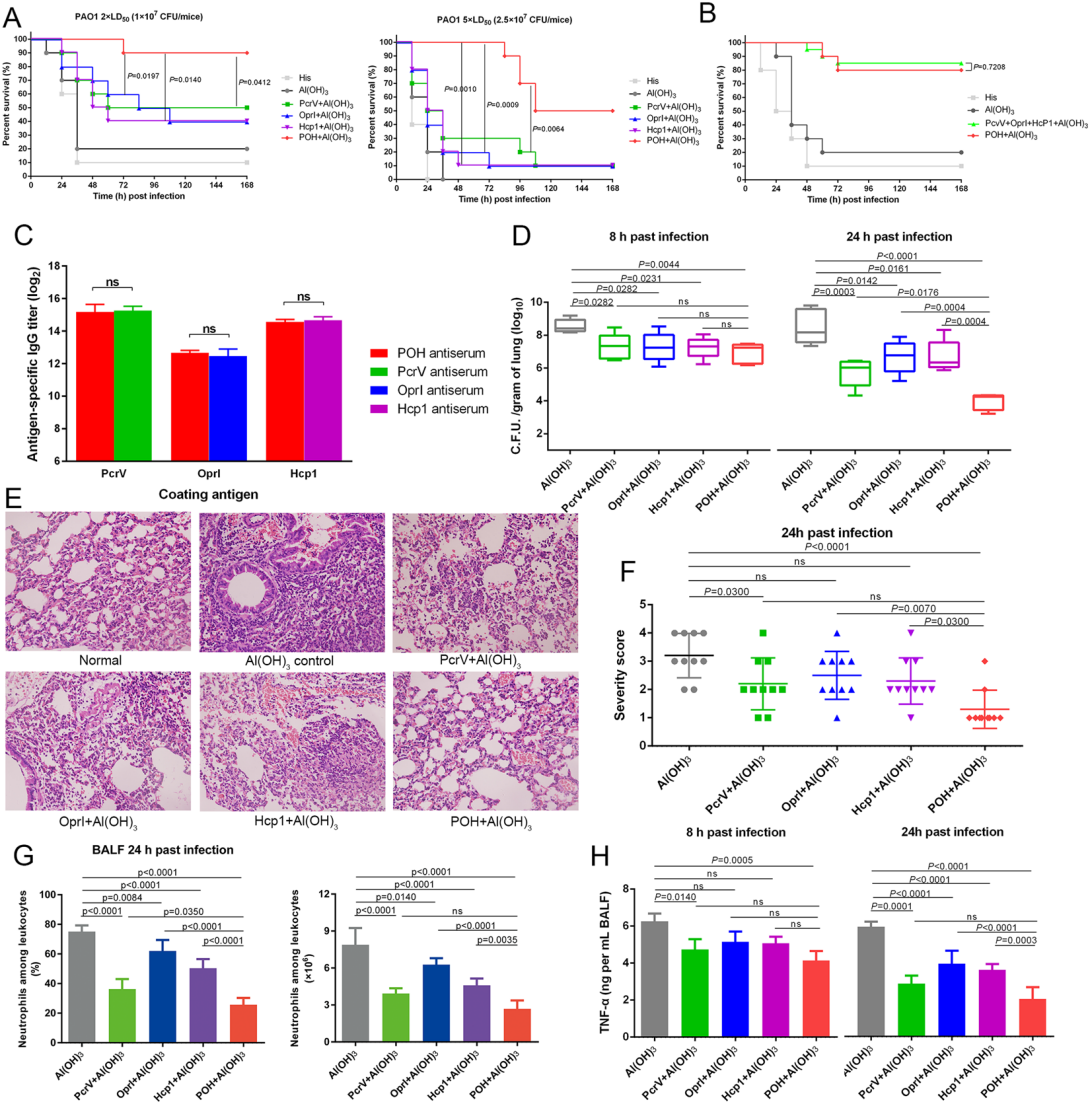
Protective efficacy of POH in murine *P*. *aeruginosa* pneumonia model. (**A**) BALB/c mice (n = 10) were immunized with antigens (PcrV, OprI, Hcp1 and POH) plus an Al(OH)_3_ adjuvant and challenged with PAO1 at 2 × LD_50_ (1.0 × 10^7^ CFUs/mouse, left panel) or 5 × LD_50_ (2.5 × 10^7^ CFUs/mouse, right panel) by intratracheal injection. The survival rate was monitored for 1 week. (**B**) BALB/c mice (n = 20) were immunized with a trivalent POH vaccine or a mixture including PcrV, OprI, and Hcp1. The mice were challenged with PAO1 at 1.0 × 10^7^ CFUs/mouse by intratracheal injection, and the survival rate was monitored for 1 week. (**C**) Plates were coated with OprI, PcrV, and Hcp1, respectively. And serum was collected from mice (n = 10) in (**A**) immunized with antigens (PcrV, OprI, Hcp1 and POH) plus Al(OH)_3_ adjuvant on day 28 before challenge. Antibody responses were expressed as the mean of log_2_ titers. Student’s t-test was used to compare values. (ns = no significance). Data were shown as the mean ± SEM. (**D**–**H**) The immunized mice and control mice were infected intratracheally with 5.0 × 10^6^ CFUs/mouse of PAO1. (**D**) The number of viable bacteria in the lungs of mice (n = 10) at 8 h and 24 h post-infection are shown. (**E**) Hematoxylin-eosin staining of lungs from immunized mice and control mice 24 hours after infection. Representative histopathological sections from 10 mice per group are shown (magnification = 200X). (**F**) Semi-quantification of lung inflammation in infected mice. Severity scores of lungs (n = 10) from immunized mice and control mice 24 hours post-infection are shown. The data are presented as scatter plots. (**G**) Evaluation of neutrophil infiltration in infected mice (n = 10). The bar represents the percentage (left) and the number (right) of neutrophils in the BALF of immunized mice (n = 10) at 24 hours post-challenge. (**H**) Quantitative detection of proinflammatory cytokines (TNF-α) in infected mice (n = 10). The data (**D**,**F**,**G** and **H**) were shown as the mean ± SD. The P-value (**A** and **B**) was calculated using the Mantel-Cox log-rank test. Multiple comparisons among different groups (**D**,**F**,**G** and **H**) were calculated using one-way ANOVA (ns = no significance).

To further confirm the protective effects of POH immunization, mice were immunized with trivalent POH and a single unit of PcrV, OprI, and Hcp1. As shown in Fig. 3A (left and right panel), at the end of the observation period, mice vaccinated with the PcrV, OprI, Hcp1 and POH both exhibited higher survival rates than the control group. And when mice challenged with 2 × LD_50_ (1.0 × 10^7^ CFUs/mouse), the trivalent POH group showed a higher protective efficacy than did vaccination with PcrV, OprI, or Hcp1 alone (*P*_POH-PcrV_ = 0.0412, *P*_POH-OprI_ = 0.0197, and *P*_POH-Hcp1_ = 0.0140, Fig. 3A left panel). Moreover, as the challenging dose reached to 5 × LD_50_ (2.5 × 10^7^ CFUs/mouse), the trivalent POH group showed a significantly higher protective efficacy than did vaccination with PcrV, OprI, or Hcp1 alone (*P*_POH-PcrV_ = 0.0064, *P*_POH-OprI_ = 0.0009, and *P*_POH-Hcp1_ = 0.0010, Fig. 3A right panel). In addition, there was no significant difference in survival between the mice immunized with trivalent POH and those immunized with a mixture of PcrV, OprI, and Hcp1 (Fig. 3B). Further, the titers of specific antibody against each antigen were not statistically different between POH and single antigen immunized mice (Fig. 3C). Therefore, vaccination with POH generated improved protective effect than a single unit of PcrV, OprI, and Hcp1.

Next, the POH-immunized mice were challenged with a sub-lethal dose of *P*. *aeruginosa* to investigate the mechanism for POH-induced protection. First, the lungs from immunized and control mice were harvested, and bacterial burden at 8 and 24 hours post-infection was evaluated. As shown in Fig. 3D, the vaccinated groups showed significantly lower bacterial loads than Al(OH)_3_ group 8 hours post-infection, but there was no difference between these four vaccinated groups. Further, the reduction in *P*. *aeruginosa* colonization was significantly enhanced in the POH group compared with that of the PcrV, OprI and Hcp1 groups 24 hours post-infection (Fig. 3D). These results suggested that POH was able to partially inhibit *P*. *aeruginosa* growth and colonization *in vivo*.

Histological analysis then showed that the lungs from the mice in the recombinant vaccine immunized groups exhibited reduced alveolar disruption, vascular leakage and deposition of bacterial microcolonies in the alveoli after infection compared with the Al(OH)_3_ group (Fig. 3E). Furthermore, typical pathological changes were observed in mice immunized with Al(OH)_3_ (Fig. 3E,F). However, mice immunized with POH exhibited reduced inflammatory cell infiltration, bleeding, and tissue damage compared with the Al(OH)_3_ group (Fig. 3E,F). Taken together, the results demonstrate that the severity of lung damage was significantly lower in the POH group than in the OprI, Hcp1 and Al(OH)_3_ groups (Fig. 3F).

Markers of inflammation including neutrophil infiltration and proinflammatory cytokine production in the BALF, such as TNF-α, IL-1β and IL-6, were determined 8 and 24 hours post-infection. Neutrophil number and percentage relative to other leukocytes was significantly reduced in the BALF of mice immunized with POH (Fig. 3G), which was in consistent with reduced cell infiltration observed by histological analysis. Despite being higher than in the POH-immunized mice, the levels of neutrophil infiltration in the PcrV-, OprI- and Hcp1-immunized mice decreased compared with that in the Al(OH)_3_ immunized mice (Fig. 3G). Similar results were observed in proinflammatory cytokine secretion (Fig. 3H and Supplementary Fig. S1). Taken together, these results confirm the protective effect of POH vaccination due to reduced bacterial burden, pathology and pro-inflammatory cytokine production.

### POH vaccination protects burned mice from *P*. *aeruginosa* infection

**Figure 4:**
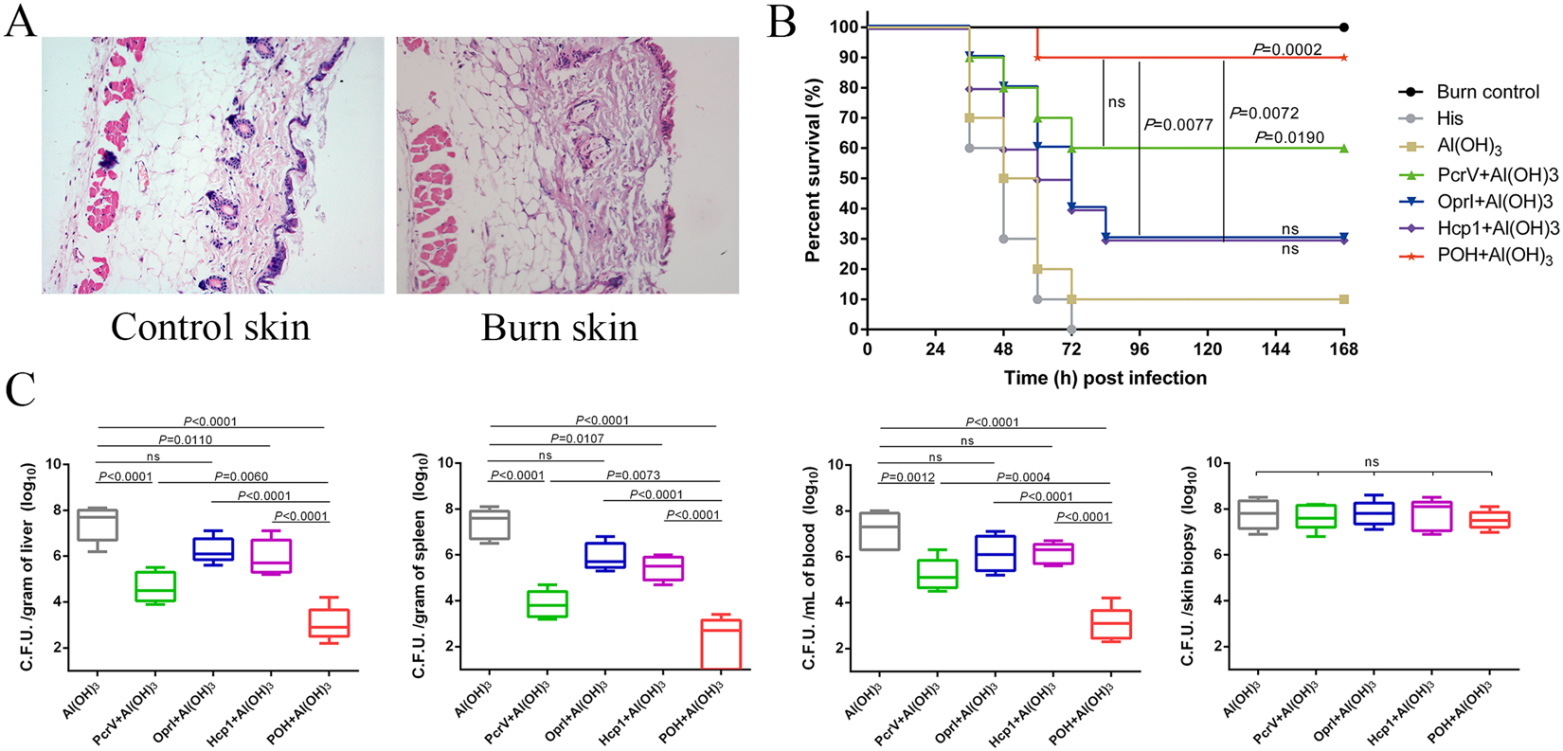
Protective effect of POH in a mouse burn model infected with *P*. *aeruginosa*. (**A**) Histopathology of the burned skin. Compared with the control unburned skin, the burn zone without nuclei included the whole epidermis and dermis (magnification = 200X). (**B**) Survival rates of immunized mice (n = 10) challenged with burn wound infections by *P*. *aeruginosa* strains PAO1. Two weeks after the final immunization (POH, PcrV, OprI, and Hcp1), the immunized mice and control mice (n = 10) were burned and subsequently challenged with PAO1 (300 CFUs for each). Survival was monitored twice daily for 7 days, and the rates were analyzed using the log-rank test method. (**C**) Effects of immunization with POH, PcrV, OprI, and Hcp1 on the local and systemic spread of *P*. *aeruginosa* strains inoculated into the burned skin. The bacterial load was determined in the liver, spleen, blood and skin (n = 10). Values represented as the mean ± SD. Differences were compared to determine their statistical significance using Student’s t-test (ns = no significance).

A third-degree burn was induced on the backs of immunized mice with hot water and was confirmed by histological examination (Fig. 4A). The skin structure was destroyed, and the damaged tissues lacked nuclei. The mice were then subcutaneously infected with *P*. *aeruginosa* to evaluate the efficacy of the POH-induced immunity. As shown in Fig. 4B, all of the mice in the unchallenged burn control group survived, the mice vaccinated with the POH, PcrV, OprI or Hcp1 exhibited higher survival rates (90%, 60%, 30%, and 30% at 168 hours post-infection, respectively) than did mice in the Al(OH)_3_ group (10% survival,) or the His group (no mice survived),and the survival rates of the POH and PcrV groups were significantly higher than that of the Al(OH)_3_ group (*P*_POH-Al(OH)3_ = 0.0002, *P*_PcrV-Al(OH)3_ = 0.0190). The trivalent POH vaccine also yielded a significantly higher protective effect than did the OprI or Hcp1 vaccines alone (*P*_POH-OprI_ = 0.0077, and *P*_POH-Hcp1_ = 0.0072), as shown in Fig. 4B.

The bacterial loads in the liver, spleen, blood, and skin were quantified to investigate the possible mechanism of the POH-induced immune response. Immunization with POH significantly decreased the bacterial burdens in the liver, spleen, and blood compared with those of the control groups (*P* < 0.0001, Fig. 4C). In addition, the bacterial loads in the skin were also decreased in the POH vaccinated mice, but these reductions were not significant compared with those in the control group (Fig. 4C). Therefore, these results strongly indicate that the trivalent POH vaccine induces protective effects in a lethal *P*. *aeruginosa* burn model.

### POH vaccination elicits Th1, Th2 and Th17 responses

**Figure 5:**
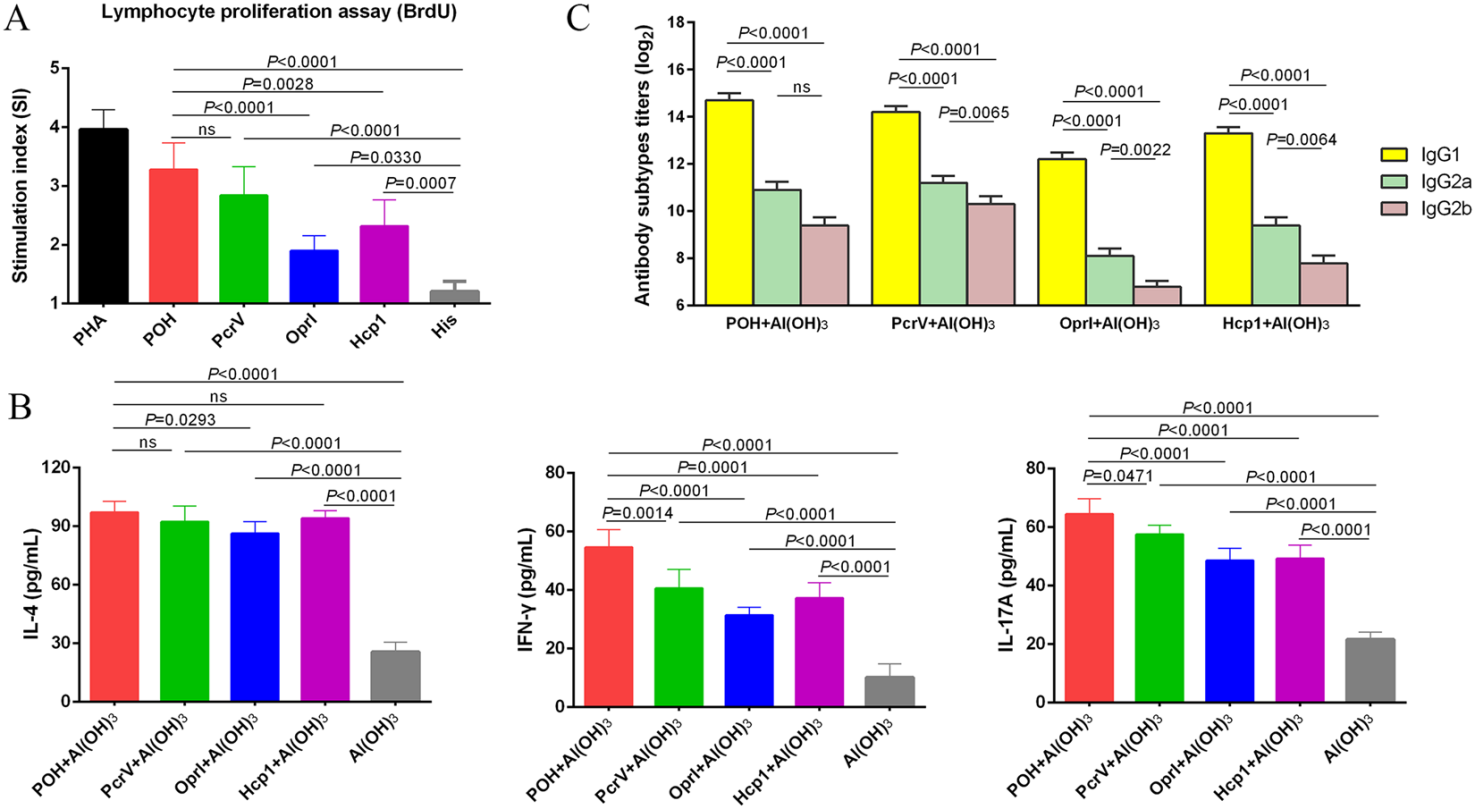
Analysis of splenocyte proliferation, cytokines and antibody responses. (**A**) Lympho-proliferative activity of mouse splenocytes (n = 5) after *in vitro* stimulation with POH, PcrV, OprI, or Hcp1 for 72 h, respectively. Proliferation was measured using the bromodeoxyuridine (BrdU) labeling method. (**B**) Comparison of cytokine production by antigens stimulated splenocytes from immunized and control mice. Two weeks after the final immunization, spleens (n = 5) were processed and stimulated with POH, PcrV, OprI, or Hcp1, and the levels of IL-4, IFN-γ, and IL-17 in each culture supernatant were measured after 72 h. (**C**) Comparison of serum IgG1, IgG2a and IgG2b subtypes among antigens in the immunized mice (n = 5). Serum was obtained at two weeks after the final immunization, and the levels of IgG1, IgG2a and IgG2b subtypes were expressed as the mean of log_2_ titers. Multiple comparisons among different groups were analyzed using one-way ANOVA (ns = no significance). Data were shown as the mean ± SEM.

To understand the POH vaccination-mediated protection, the immune response in the spleen was also evaluated in addition to assessing serum antibodies. First, the stimulation index of splenocytes from mice immunized with POH, PcrV, OprI or Hcp1 was analyzed. As shown in Fig. 5A, the stimulation indices of mouse splenocytes treated with POH, PcrV, OprI or Hcp1 were all significantly higher than that of the Al(OH)_3_ control group (*P*_POH_ < 0.0001, *P*_PcrV_ < 0.0001, *P*_OprI_ = 0.0330, *P*_Hcp1_ = 0.0007). The mice immunized with POH achieved the highest splenocyte lympho-proliferation among the four groups, and this proliferation was significantly higher than that of OprI or Hcp1 alone (*P*_POH-OprI_ < 0.0001, and *P*_POH-Hcp1_ = 0.0028) (Fig. 5A). However, there was no difference between the POH and the PcrV group (Fig. 5A).

The lymphocyte proliferation rates of splenocytes from mice immunized with POH, PcrV, OprI or Hcp1 were consistent with SI (Supplementary Table S4 and Figure S2). And the proliferation of different phenotypes (CD3^−^CD19^+^, CD3^+^CD4^+^, CD3^+^CD8^+^ cells) were observed with different extent. Notably, the ratio of CD4^+^/CD8^+^ T cells in all proteins immunization groups increased significantly compared with that of the control group (*P* < 0.05), which showed that the CD4^+^ T cell population was predominant in the proliferative T cells (Supplementary Table S4).

Next, the cytokine levels in the supernatants were measured after stimulation with recombinant proteins. The splenocytes from the vaccinated mice produced significantly more IL4, IFN-γ and IL-17A when stimulated with the corresponding recombinant proteins than did the Al(OH)_3_ control group (*P* < 0.0001, Fig. 5B). Furthermore, the splenocytes from the POH group produced the greatest amounts of cytokines (Fig. 5B), demonstrating that splenocytes from mice immunized with POH could produce more IL4, IFN-γ and IL-17A following antigen encounters.

In addition, to determine the type of antibody response induced by the recombinant proteins, the levels of IgG subgroups (IgG1, IgG2a and IgG2b) in the immunized mouse sera were also measured. As shown in Fig. 5C, POH induced higher levels of antigen-specific IgG1, IgG2a and IgG2b subtypes compared with those in the Al(OH)_3_ control group (*P*_IgG1_ < 0.0001, *P*_IgG2a_ = 0.0003 and *P*_IgG2b_ = 0.0001). Furthermore, the level of antigen-specific IgG1 was approximately 160 times higher than IgG2a (Fig. 5C). IgG1 and IgG2a are markers for Th2 and Th1 responses, respectively. These results suggest that a Th2-biased response was induced, which is consistent with the trend of cytokine production in the supernatants.

### POH specific antibodies are functional *in vitro* and *in vivo*

**Figure 6:**
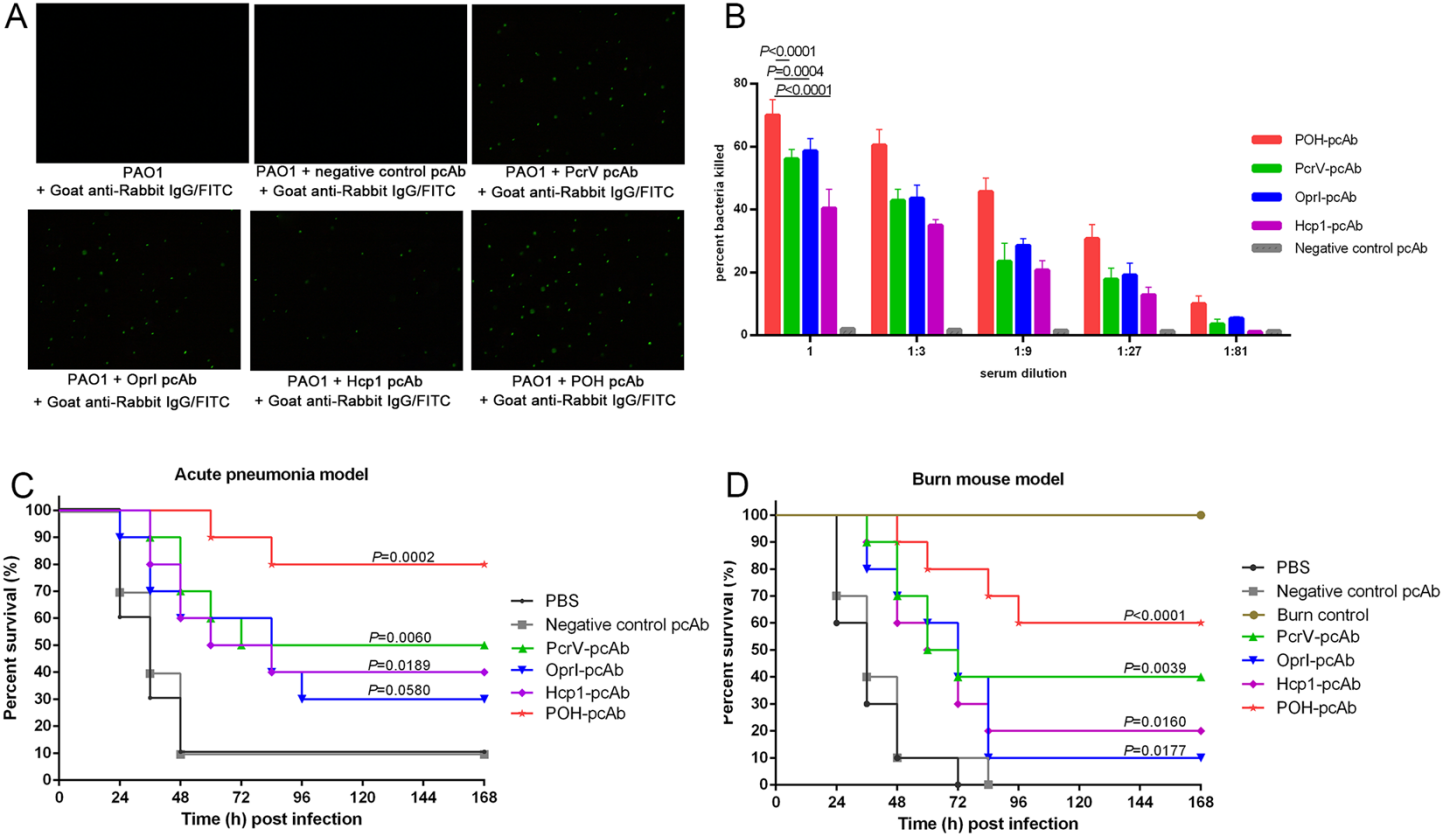
Antigen-induced humoral immune response is essential for POH-mediated protection. (**A**) pcAbs are able to directly recognize PAO1 *in vitro*. Indirect immunofluorescence was conducted to confirm the binding of pcAbs from rabbits immunized with POH, PcrV, OprI, or Hcp1 to PAO1 *in vitro*. No immunofluorescence was detected in the absence of pcAb. Positive, indirect immunofluorescence signals indicated binding of different pcAb with PAO1. This study was performed twice, with similar results. (**B**) Comparative analysis of opsonophagocytic killing activity against *P*. *aeruginosa* strains PAO1 by pcAbs. PAO1 was incubated in the presence of isolated HL-60 cells, pcAb and infant rabbit complement for 90 min at 37 °C and was then plated on agar medium and incubated for 20 h. The percentage of killing was calculated to determine killing activity. The data shown are the mean ± standard deviation (SD) derived from three independent experiments. The differences between each group are presented as a p-value, which was calculated by one-way ANOVA. (**C**) Passive immunization with pcAbs protected mice from lethal *P*. *aeruginosa* challenge in the mouse pneumonia model. Mice (n = 10) were injected intravenously with 100 μl of each pcAb (20 mg/ml). Two hours later, all of the mice were challenged with strain PAO1 at 1.0 × 10^7^ CFUs/mouse by intratracheal injection, and mouse survival was monitored for 1 week. (**D**) Passive immunization with pcAbs protected mice from lethal *P*. *aeruginosa* challenge in the mouse burn model. Mice (n = 10) were injected intravenously with 100 μl of each pcAb (20 mg/ml). Two hours later, immunized mice and control mice (n = 10) were burned and subsequently challenged with PAO1 (300 CFUs/mouse). Survival was monitored twice daily for 1 week. The Mantel-Cox log-rank test was used to compare differences between groups (**B** and **C**).

Indirect immunofluorescence assay was carried out to validate the interaction between pcAb and PAO1. The results clearly indicated that no immunofluorescence was detected in the absence of antigen specific pcAbs, whereas positive indirect immunofluorescence signals indicated the binding of four kinds of pcAb with PAO1 (Fig. 6A). These results indicated that all pcAbs generated in this study were able to bind PAO1 *in vitro*, and the binding of POH-pcAb with PAO1 may essential in clearing and controlling the infection.

To investigate whether the POH-specific antibodies were independently protective, an OPA that measured antibody- and complement-mediated bacterial killing was performed *in vitro*. The killing assay was performed using immune cells (neutrophils) that play a critical role in the host clearance of *P*. *aeruginosa*. In the presence of HL60 phagocytic cells and complement, all four pcAbs exhibited significantly increased opsonophagocytic activity for *P*. *aeruginosa* (*P* < 0.0001, Fig. 6B), the percentage of killed *P*. *aeruginosa* in the POH-pcAb group (70.3%) was significantly higher than that in the PcrV-pcAb group (56.1%, *P*_POH-PcrV_ < 0.0001), OprI-pcAb group (58.6%, *P*_POH-OprI_ = 0.0004), and Hcp1-pcAb group (40.4%, *P*_POH-Hcp1_ < 0.0001, Fig. 6B). These results indicate that the POH-specific antibodies were the most effective at killing *P*. *aeruginosa*. Thus, the antibody response may functionally regulate phagocytosis and killing of *P*. *aeruginosa* by innate immune cells.

Then, POH-specific antibody-mediated protection was verified *in vivo* by passive immunization in an acute pneumonia model and a mouse burn model. As shown in Fig. 6C, mice immunized with POH-pcAb, PcrV-pcAb, OprI-pcAb, and Hcp1-pcAb all displayed higher survival rates (80%, 50%, 30% and 40%, respectively) than did the PBS control group (10% survival) in the acute pneumonia model. The mice that were passively immunized with POH-pcAb exhibited the highest survival rate, which was significantly higher than that of any other group (*P* < 0.05, Fig. 6C). For the other three experimental groups immunized with antibodies generated using only a single antigen, the mice immunized with PcrV-pcAb exhibited the highest survival rate when compared with mice immunized with either OprI-pcAb or Hcp1-pcAb (Fig. 6C). In addition, passive immunization extended the survival time of mice, in the PBS control group, the mice did not survive more than 2 days. In contrast, the mean survival time of the immunized mice was approximately 4 days (Fig. 6C). In addition, similar survival results were observed in the mouse burn model (Fig. 6D). Notably, a higher survival rate was not observed in mice that were immunized with control pcAb compared with that of mice immunized with PBS (Fig. 6C,D), which indicates that the protection efficacy observed was directly provided by antigen-specific pcAbs.

### POH vaccination protects mice against clinical *P*. *aeruginosa* strains in murine pneumonia and burn models

**Figure 7:**
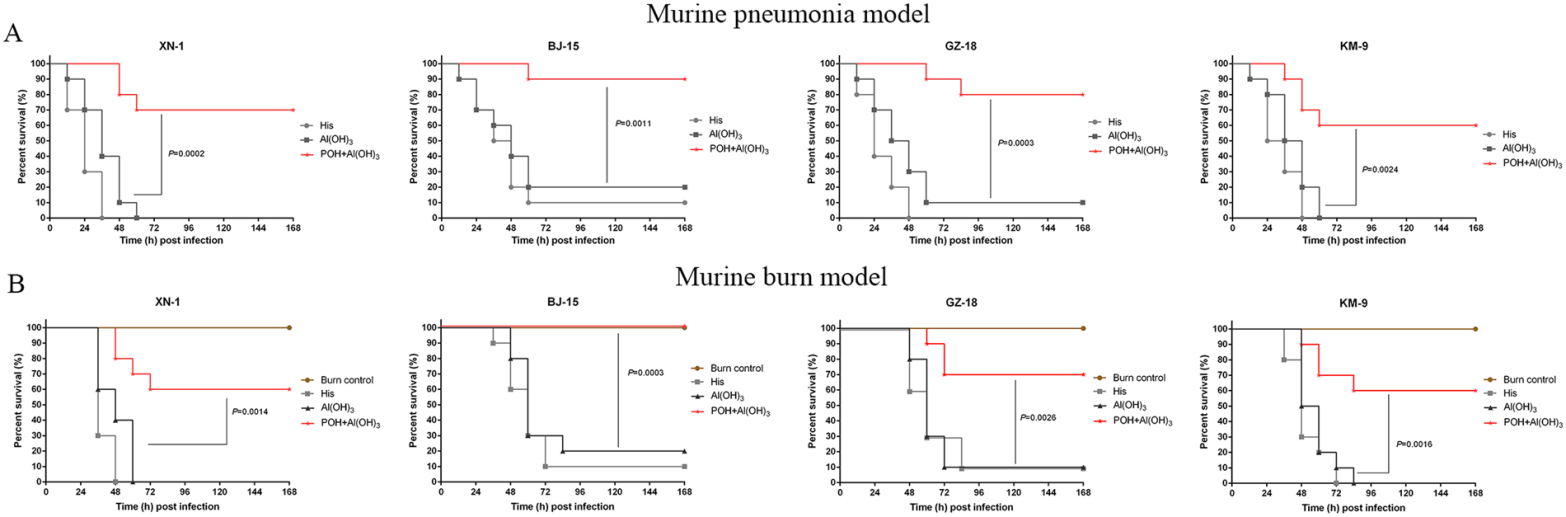
Mice immunized with POH broadly protected mice from different *P*. *aeruginosa* clinical strains challenge in murine pneumonia and burn models. (**A**) BALB/c mice (n = 10) immunized with POH were challenged by intratracheal injection with various *P*. *aeruginosa* clinical strains XN-1 (6.0 × 10^6^ CFUs), BJ-15 (2.0 × 10^7^ CFUs), GZ-18 (2.0 × 10^7^ CFUs) and KM-9 (4.0 × 10^6^ CFUs). Survival rates were monitored twice daily for 1 week. The Mantel-Cox log-rank test was used to compare differences between groups. (**B**) Comparison of survival rates of Al(OH)_3_-injected, POH-immunized (n = 10) mice that were challenged with burn wound infections by *P*. *aeruginosa* clinical isolates XN-1, BJ-15, GZ-18 and KM-9 (300 CFUs) at the burn site. Survival was monitored twice daily for 1 week, and the survival rates were analyzed using the log-rank test method.

To determine whether POH could provide broad protection, immunized mice were challenged with four different clinical strains of *P*. *aeruginosa*. The four clinical strains were chosen from a library established in our lab based on their representation and diversity. The library contains more than 400 clinical isolates collected from different districts in China. Information regarding the 4 isolates is listed in Supplementary Table S1. As shown in Fig. 7A, all 4 clinical strains exhibited different pathogenicity and virulence in mice, as indicated by the survival rates in the His control groups. POH was able to protect 60% to 70% of mice from the clinical isolate challenge when compared with the Al(OH)_3_ controls (*P*_XN-1_ = 0.0002, *P*_BJ-15_ = 0.0011, *P*_GZ-18_ = 0.0003, *P*_KM-9_ = 0.0024, Fig. 7A). In addition, the four clinical strains exhibited different pathogenicity and virulence in the burn mice, as shown by the survival rates in the His control groups (Fig. 7B). POH was able to protect 50% to 80% of mice from the clinical isolate challenge when compared with the Al(OH)_3_ controls (*P*_XN-1_ = 0.0014, *P*_BJ-15_ = 0.0003, *P*_GZ-18_ = 0.0026 and *P*_KM-9_ = 0.0016), as shown in Fig. 7B.

## Discussion

Antigens are critical for the development of effective vaccines. In this study, three antigens were selected for vaccine development against *P*. *aeruginosa* infection for the following reasons. First, the immunogenicity and protective efficacy of OprI and PcrV have been well studied elsewhere. Both active immunization with OprI^34, 35^ and passive immunization with PcrV^14, 36–39^ can protect animals from fatal *P*. *aeruginosa* challenge in different animal models, and the efficacy of both agents have already been evaluated in clinical trials^8, 40, 41^. Hcp1 was a new candidate antigen screened by our lab using reverse immunology. It exhibited strong immunogenicity in mice and was able to partially protect mice form *P*. *aeruginosa* challenge. Second, the sequences of all three antigens were highly conserved among different *P*. *aeruginosa* isolates, and therefore, they may provide cross-protective efficacy during challenge with different clinical strains. Third, single target vaccines may be inadequate for inducing an effective immune response. As shown in this study, immunization with any of the three antigens resulting in lower than 50% of protection. However, higher than 80% of protection was observed when immunization with the 3 proteins together or as a fusion protein.

Interestingly, a number of publications have focused on the protective efficacy of polyclonal or monoclonal antibodies against PcrV during *P*. *aeruginosa* infection^14, 36–39^, but few reports have described vaccines based on this antigen. We speculate that this may be attributed to the pool solubility of this protein. In our previous work, a number of PcrV constructs were designed and cloned into different vectors with different tags, only a truncated form of PcrV (Glutamic_28_-Isoleucine_294_) was partially soluble expressed in vector pGEX-6p-2 with a N-terminal GST tag, and this protein was soluble after cleaving the GST tag. Similarly, when designing the trivalent protein containing the three antigens, we also found that both the order of these proteins and the linker have a significant impact on the expression and solubility of the fusion protein. Only three proteins in a tandem sequence of POH can be expressed as soluble proteins, and the other two proteins and the linkers may facilitate the expression and folding of PcrV. Further, as shown in Fig. 3C, the titer of antigen specific antibodies induced by POH was similar to that induced by the 3 proteins, respectively. This result indicated that POH immunization would not cause interference among each vaccine component in induction of protective antibodies.

The question as to what type of immunity is essential for an effective *P*. *aeruginosa* vaccine is still controversial. Early studies emphasized the importance of high levels of opsonophagocytic antibodies in the clearance of the infection. For example, antibodies against alginate have been shown to correlate with less *P*. *aeruginosa* infection in CF patients^42^, and passive immunization with pcAb or MAbs against *P*. *aeruginosa* antigens have already shown potential in the treatment of acute *P*. *aeruginosa* pneumonia^43^. However, more recent studies have suggested that antibodies alone are insufficient to protect against *P*. *aeruginosa* infection, and it seems that multiple cellular and humoral facets of the immune system must be simultaneously activated to provide protection. Some studies have described a Th1-dominated response, independent of antibody production that plays a protective role during chronic *P*. *aeruginosa* pneumonia^44–46^. Another study also reported that PopB-immunized mice were protected from lethal pneumonia in an antibody-independent, IL-17-dependent manner^47^. In addition, some other immune system components, such as neutrophils^48^, macrophages^49^, complement^50^, NKT cells^51^ and Th22 cells^52^, have also been reported to provide protection against *P*. *aeruginosa* infection.

In this study, antigen specific Th1, Th2, and Th17 cell responses in the spleen and antibodies in the serum were observed in POH immunized mice, thus the comprehensive immune response to vaccination could be the reason for the effective POH-induced protection. However, this raised the question that which type of immune response was pivotal to POH-induced protection. In this study, opsonophagocytic killing assay showed that *P*. *aeruginosa* could be killed by POH specific antibody efficiently *in vitro*. Besides, passive immunization with POH specific antibodies resulting in 80% of protection against *P*. *aeruginosa* infection in mice, and the protection was obtained in a dose dependent manner (data not shown). These results suggesting that antibody is critical in POH mediated protection, and this was in consistence with former reports that OprI- and PcrV-specific antibodies were critical for protection against *P*. *aeruginosa* infection^13, 35^. Moreover, protection was also observed in the burn model in this study, as the disorder of immune function followed the tissue damage due to the burn, this protection was mainly provided by antibodies induced before the burn. On the other hand, up to 90% of protection was observed in the pneumonia model by active immunization, which was slightly higher than that observed in passive immunization, this difference indicated that cellular immune response was also contributed to the protection induced by POH immunization.

Furthermore, due to the conservation of the sequences of the three antigens, cross-protective efficacy was observed among different clinical isolates of *P*. *aeruginosa*. These results indicated that POH is a promising antigen for the development of a vaccine against *P*. *aeruginosa*. Typically, nasal immunization is considered to be the first choice for immunization against mucosal infection. In the current study, although intramuscular immunization was used in the pneumonia model, high protective efficacy accompanied by a lower bacterial burden and inflammatory cell infiltration was also observed. This may be explained by the adequate blood supply to the lung tissue, suggesting that intramuscular immunization can be used as an alternative route for immunization against pneumonia.

Taken together, our results showed that immunization with the trivalent candidate antigen (POH) designed in this study significantly reduced acute skin infection and pneumonia due to *P*. *aeruginosa* in mice. Furthermore, passive immunization suggested that humoral immune responses play a dominant role in POH-mediated protection. Moreover, POH immunization induced a cross-protective efficacy among different clinical isolates of *P*. *aeruginosa*. Therefore, POH can be regarded as a novel *P*. *aeruginosa* vaccine candidate. Because *P*. *aeruginosa* has posed therapeutic challenges worldwide as some strains have become resistant to nearly all front-line antibiotics, our results provided the basis for an alternative strategy for the prevention of *P*. *aeruginosa* infection by immunization.

## Electronic supplementary material

Supplementary information
