# Supplementary material for: Protective Efficacy of the Trivalent Pseudomonas aeruginosa Vaccine Candidate PcrV-OprI-Hcp1 in Murine Pneumonia and Burn Models
Source: Sci Rep. 2017 Jun 21;7:3957. doi: 10.1038/s41598-017-04029-5 (PMC5479855; doi:10.1038/s41598-017-04029-5)

Protective Efficacy of the Trivalent *Pseudomonas aeruginosa* Vaccine Candidate PcrV-OprI-Hcp1 in Murine Pneumonia and Burn Models

Feng Yang[1, 2, +], Jiang Gu [2, +], Liuyang Yang[2], Chen Gao[2], Haiming Jing[2], Ying Wang[2], Hao Zeng[2], Quanming Zou[2], Fenglin Lv[1, *] & Jinyong Zhang[2, *]

[1]College of Bioengineering, Chongqing University, Chongqing, 400030, PR China

[2]National Engineering Research Center of Immunological Products, Department of Microbiology and Biochemical Pharmacy, College of Pharmacy, Third Military Medical University, Chongqing, 400038, PR China.

[+] These authors contribute equally to this work.

[*]Corresponding author:

Fenglin Lv, Chongqing shapingba district 174 shazheng street, Chongqing, 400030, PR China; Fax: 86-23-68752377, E-mail: lyufenglin@cqu.edu.cn

Jinyong Zhang, Chongqing shapingba district 30 gaotanyan street, Chongqing, 400038, PR China; Fax: 86-23-89866779, E-mail: zhangjy198217@126.com

Supplementary Table S1. The information of *P. aeruginosa* clinical strains used in this study.

Strain name	Source of strain	Department	Patient's name	Gender	Age	Source of sample	Serological typing [&]
XN-1	Chongqing southwest hospital	Respiratory	Wang××	Female	69	Sputum	B
BJ-15	Beijing 263 hospital	ICU	Yang×	Male	41	Blood	G
GZ-18	Guangzhou nanfang hospital	ICU	Zhang×	Female	31	Sputum	E
KM-9	Kunming medical college	Burns surgery	Li××	Male	6	Secretions	I

[&] Serotypes of *P. aeruginosa* isolated strains were detected by P. aeruginosa serotyping kit (Nihon Seikagaku Kenkyujo Co., Japan), based on O-group antigens of *P. aeruginosa*.

Supplementary Table S2. The primers used in this study.

Gene	The forward primer	The reverse primer
PcrV	5’-CGCGGATCCGAGCAGGAGGAACTGCTGG-3’	5’-TTTTCCTTTTGCGGCCGCCTAGATCGCGCTG-3’
OprI	5’-CGCGGATCCAAAGAAACCGAAGCTCGTCTG-3’	5’-TTTTCCTTTTGCGGCCGCTTACTTGCGGCTG-3’
Hcp1	5' CGCGGATCCATGGCTGTTGATATGTTCATC-3'	5'-CCGCTCGAGTCAGGCCTGCACGTTCTGGCGGATG-3'

Supplementary Table S3. The POH DNA and amino acid sequences.

POH	sequences
DNA	GGATCCGAGCAGGAGGAACTGCTGGCCCTGTTGCGCAGCGAGCGGATCGTGCTGGCCCAC<br>GCCGGCCAGCCGCTGAGCGAGGCGCAAGTGCTCAAGGCGCTCGCCTGGTTGCTCGCGGCC<br>AATCCGTCCGCGCCTCCGGGGCAGGGCCTCGAGGTACTCCGCGAAGTCCTGCAGGCACGT<br>CGGCAGCCCGGTGCGCAGTGGGATCTGCGCGAGTTCCTGGTGTCGGCCTATTTCAGCCTGC<br>ACGGGCGTCTCGACGAGGATGTCATCGGTGTCTACAAGGATGTCCTGCAGACCCAGGACG<br>GCAAGCGCAAGGCGCTGCTCGACGAGCTCAAGGCGCTGACCGCGGAGTTGAAGGTCTAC<br>AGCGTGATCCAGTCGCAGATCAACGCCGCGCTGTCGGCCAAGCAGGGCATCAGGATCGAC<br>GCTGGCGGTATCGATCTGGTCGACCCCACGCTATATGGCTATGCCGTCGGCGATCCCAGGTG<br>GAAGGACAGCCCCGAGTATGCGCTGCTGAGCAATCTGGATACCTTCAGCGGCAAGCTGTC<br>GATCAAGGATTTTCTCAGCGGCTCGCCGAAGCAGAGCGGGGAACTCAAGGGCCTCAGCGA<br>TGAGTACCCCTTCGAGAAGGACAACAACCCGGTCGGCAATTTCGCCACCACGGTGAGCGA<br>CCGCTCGCGTCCGCTGAACGACAAGGTCAACGAGAAGACCACCCTGCTCAACGACACCA<br>GCTCCCGCTACAACTCGGCGGTCGAGGCGCTCAACCGCTTCATCCAGAAATACGACAGCG<br>TCCTGCGCGACATTCTCAGCGCGATCGGTGGTGGTGGTTCAAAAGAAACCGAAGCTCGTC<br>TGACCGCTACCGAAGACGCAGCTGCTCGTGCTCAGGCTCGCGCTGACGAAGCCTATCGCA<br>AGGCTGACGAAGCTCTGGGCGCTGCTCAGAAAGCTCAGCAGACTGCTGACGAGGCTAAC<br>GAGCGTGCCCTGCGCATGCTGGAAAAAGCCAGCCGCAAGGGTTCTGGAGGATCAGGTATG<br>GCTGTTGATATGTTCATCAAGATCGGCGACGTCAAGGGTGAGTCCAAGGACAAGACTCAC<br>GCCGAGGAAATCGACGTGCTGGCATGGAGCTGGGGCATGTCCCAGTCCGGGTCGATGCAC<br>ATGGGCGGTGGCGGCGGCGCCGGCAAGGTCAACGTGCAGGACCTGTCGTTCACCAAGTAC<br>ATCGACAAGTCCACGCCCAACCTGATGATGGCCTGCTCCAGCGGCAAGCACTATCCGCAGG<br>CGAAGCTGACCATCCGCAAGGCCGGCGGCGAGAACCAGGTCGAGTACCTGATCATCACCC<br>TGAAGGAAGTCCTGGTGTCCTCGGTGAGCACCGGCGGCAGCGGTGGCGAGGATCGCCTGA<br>CCGAGAACGTGACCCTGAACTTCGCCCAGGTCCAGGTCGACTACCAGCCGCAGAAGGCGG<br>ATGGCGCGAAGGACGGCGGTCCGGTCAAGTACGGCTGGAACATCCGCCAGAACGTGCAG<br>GCCTGA
amino acid	GSEQEELLALLRSERIVLAHAGQPLSEAQVLKALAWLLAANPSAPPGQGLEVLREVLQARRQP<br>GAQWDLREFLVSAYFSLHGRLDEDVIGVYKDVLQTQDGKRKALLDELKALTAELKVYSVIQS<br>QINAALSAKQGIRIDAGGIDLVDPTLYGYAVGDPRWKDSPEYALLSNLDTFSGKLSIKDFLSGSP<br>KQSGELKGLSDEYPFEKDNNPVGNFATTVSDRSRPLNDKVNEKTTLLNDTSSRYNSAVEALNR<br>FIQKYDSVLRDILSAIGGGGSKETEARLTATEDAAARAQARADEAYRKADEALGAAQKAQQT<br>ADEANERALRMLEKASRKGSGGSGMAVDMFIKIGDVKGESKDKTHAEEIDVLAWSWGMSQS<br>GSMHMGGGGGAGKVNVQDLSFTKYIDKSTPNLMMACSSGKHYPQAKLTIRKAGGENQVEY<br>LIITLKEVLVSSVSTGGSGGEDRLTENVTLNFAQVQVDYQPQKADGAKDGGPVKYGWNIRQN<br>VQA

Supplementary Table S4. The rates of proliferation in total and different phenotype determined by flow cytometer.

Group	Proliferating cells ((%)				Ratio
	Total	$CD3^-CD19^+$	$CD3^+CD4^+$	$CD3^+CD8^+$	$CD4^+/CD8^+$
PHA	80.68 ± 6.26	32.68 ± 3.35	6.94 ± 1.32	35.50 ± 2.89	0.20 ± 0.07
POH	60.28 ± 6.02 [a]	37.61 ± 4.68 [a]	16.82 ± 2.28 [a]	2.46 ± 0.35 [a]	6.84 ± 0.76 [a]
PcrV	41.65 ± 4.78 [a]	25.74 ± 2.36 [a]	11.01 ± 1.65 [a]	1.98 ± 0.46 [a]	5.55 ± 0.43 [a]
OprI	20.38 ± 2.97 [a]	12.41 ± 1.66 [a]	5.81 ± 0.21 [a]	0.89 ± 0.25 [a]	6.55 ± 0.31 [a]
Hcp1	32.15 ± 3.75 [a]	19.10 ± 2.21 [a]	8.29 ± 1.65 [a]	1.71 ± 0.32 [a]	4.86 ± 0.09 [a]
His	2.69 ± 0.73	1.67 ± 0.31	0.29 ± 0.11	0.44 ± 0.14	0.67 ± 0.83

[a] The difference of means between the immunization group and the negative control group is significant at the 0.05 level.

Supplementary Fig. S1. Quantitative detection of IL-1β and IL-6.

The immunized mice and control mice were infected intratracheally with 5.0×10^6 CFU/mouse of PAO1. Proinflammatory cytokines (IL-1β and IL-6) in the BALF of infected mice (n = 10) were detected 8 and 24 hours post-infection. The data are shown as the mean ± SD. Multiple comparisons among different groups were calculated using one-way ANOVA (ns = no significance).

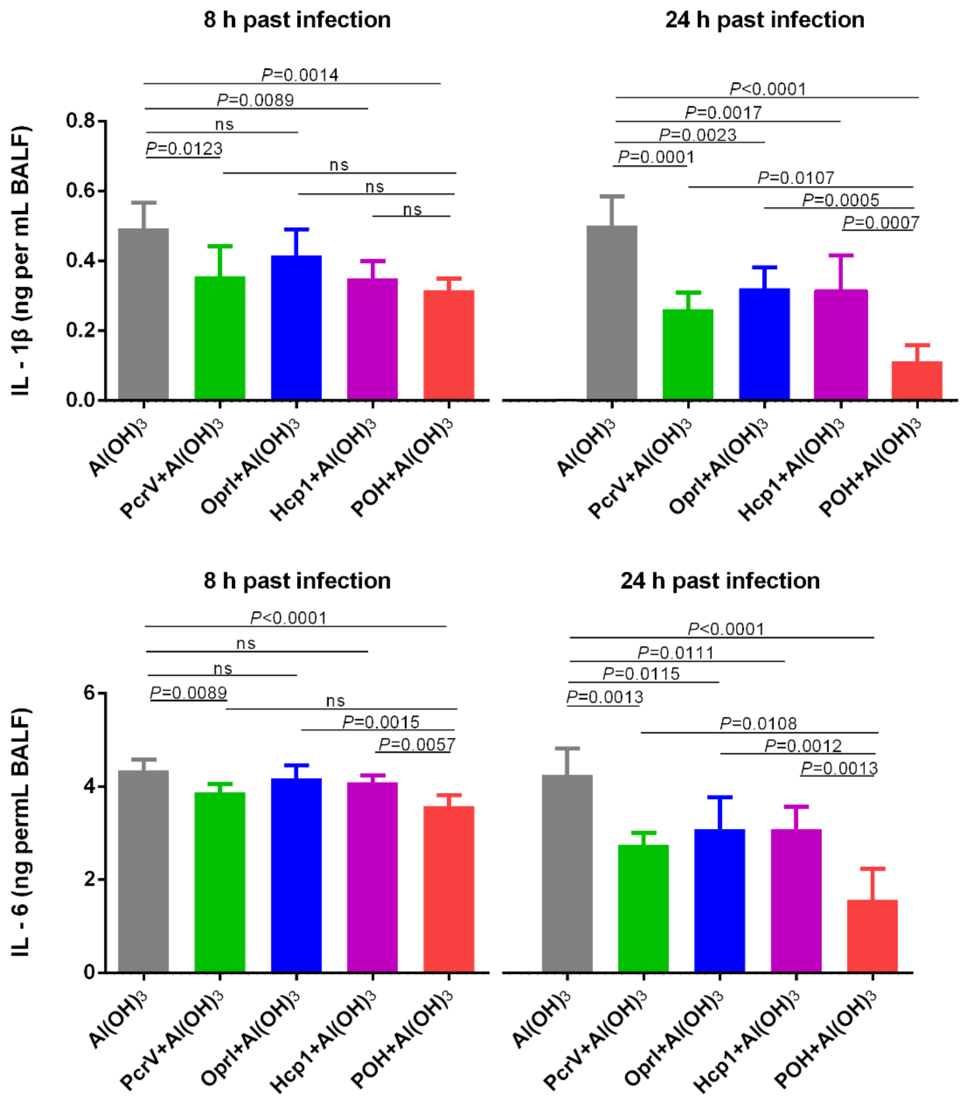

Supplementary Fig. S2. Representative flow cytometry results of splenocyte proliferative response.

Splenocytes from immunized mice were incubated with CFSE, and then stimulated with the corresponding antigen POH, PcrV, OprI or Hcp1. Cells treated with 10 mM phytohemagglutinin-A (PHA) (Gibco, USA) or not served as positive and negative controls, respectively.

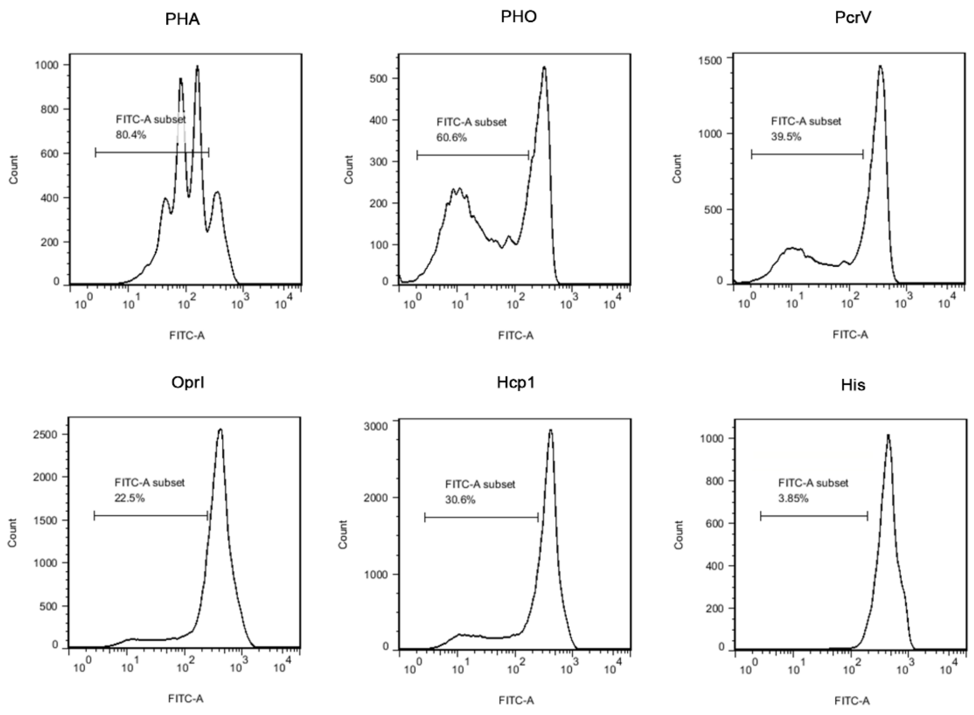

Supplement: Supplementary file 1 — Supplementary information [file 41598_2017_4029_MOESM1_ESM.pdf]
